# Characterization of Phenotypes of Immune Cells and Cytokines Associated with Chronic Exposure to *Premolis semirufa* Caterpillar Bristles Extract

**DOI:** 10.1371/journal.pone.0071938

**Published:** 2013-09-04

**Authors:** Isadora Maria Villas-Boas, Rute Maria Gonçalves-de-Andrade, Carla Cristina Squaiella-Baptistão, Osvaldo Augusto Sant'Anna, Denise V. Tambourgi

**Affiliations:** Immunochemistry Laboratory, Butantan Institute, São Paulo, SP, Brazil; Centro de Investigacion y de Estudios Avanzados del Instituto Politecnico Nacional, Mexico

## Abstract

The Brazilian moth *Premolis semirufa* (Walker, 1856), usually called pararama, is a parasite of the rubber *Hevea* genus. Contact with the bristles causes symptoms of acute inflammation. A chronic inflammatory reaction frequently occurs in individuals after multiple contacts, and this reaction is characterised by articular synovial membrane thickening with joint deformities, common characteristics of chronic synovitis. Extract from the bristles has been shown to induce an intense inflammatory response in a murine model, and this reaction was characterised by the presence of neutrophils in the paw tissues of injected mice and a strong, specific antibody response. There is not yet an effective treatment for incidents involving contact with pararama. In this study, we evaluated the phenotype of the immunological response and cytokine production in BALB/c mice subcutaneously injected in the footpad with *P*. *semirufa* bristle extract or sterile saline (control) seven times at 15 day intervals. An analysis of cells from the draining lymph node by flow cytometry showed that the absolute numbers of TCD4, TCD8 and B lymphocytes, as well as the expression of activation molecules, were higher in the extract-treated group. Furthermore, immunohistochemistry and immunofluorescence analyses showed a mixed inflammatory infiltrate composed of neutrophils and macrophages at the inoculation site. In addition, an analysis of paw cytokines showed elevated levels of IL-6, IL-12, IL-10, IL-17 and IL-23 after the 7^th^ inoculation. In conclusion, these data provide evidence of pro-inflammatory changes in the phenotypes of immune cells and cytokine production in animals subjected to injections with an extract from *Premolis semirufa* bristles, which may explain the intense and prolonged inflammatory response that characterises this disorder.

## Introduction

The Brazilian moth *Premolis semirufa* (Walker, 1856), usually called pararama in its larval stage, belongs to the Arctiidae family and inhabits rubber plantations in the Amazon region, feeding of the rubber tree *Hevea brasiliensis*. The tappers, when collecting the latex, can stick their fingers in the trunk of the rubber trees to facilitate the harvest and, at that time, may come into contact with the pararama. Known as “Pararama associated phalangeal periarthritis” and due to its importance as an occupational disease, predominantly in the rubber tree areas of Pará, Brazil, this caterpillar envenomation was included into the “Manual of diagnosis and treatment of envenomations”, by the Brazilian Ministry of Health in 1992 [1].

The contact with the pararama caterpillar bristles generally causes an instant intense itching followed by symptoms of acute inflammation, such as pain, heat and redness, which last up to seven days after the first incident [2], [3]. Chronic symptoms, which frequently occur in individuals after multiple contacts are characterised by synovial membrane thickening, with joint deformities and chronic synovitis (mono- or oligoarticular) that may progress to joint immobility [4].

The disease caused by contact with the bristles of *Premolis semirufa* shares many features with the symptoms of inflammatory joint disease, unlike the clinical manifestations presented by the venom of other caterpillars, such as the erythema, kidney and liver damage caused by *Dirphia* sp. (Saturniidae) [5], [6]; allergic reactions induced by contact with *Euproctis chrysorrhea* (Lymantriidae) [7]; and homeostatic abnormalities such as blood coagulation and fibrinolysis, as well as bleeding through the mucous membranes and internal organs including the brain (which may lead to death), caused by contact with *Lonomia* sp. (Saturniidae) [8], [9].

The most common form of the inflammatory joint disease is rheumatoid arthritis (RA), a chronic, systemic disorder that causes inflammation in the synovium [10], [11]. In this disease, CD4^+^ T cells, B cells and macrophages infiltrate the synovium, where they are activated and contribute to local destruction. Additionally, neutrophils accumulate in the synovial fluid, where they engulf immune complexes and release proteolytic enzymes. Furthermore, a broad array of macrophage and fibroblast cytokines, including interleukin (IL)-1, IL-6, IL-15, IL-18, tumour-necrosis factor (TNF)-α, granulocyte-macrophage colony-stimulating factor (GM-CSF), various chemokines, and many others, are produced by the rheumatoid synovium. These cytokines, as well as proteases that contribute to cartilage destruction, perpetuate the inflammation [12]. Small but physiologically relevant amounts of IFN-γ and IL-17 cytokines are expressed in RA, which may contribute to immune responses, fibroblast activation and bone destruction [13].

Despite its similarity with some inflammatory joint features, the disease caused by contact with *Premolis semirufa* does not seem to be a systemic autoimmune disorder because it does not induce the generation of autoantibodies, such as anti-DNA or anti-collagen type II, as shown in our previous study. Moreover, we have demonstrated that *Premolis semirufa* caterpillar bristles' crude extract presents strong proteolytic activity. We observed that the bristles' extract can induce an intense inflammatory process, characterised by the presence of neutrophils in the paw tissues of injected mice and a strong, specific antibody response [14].

To better understand the participation of the elements of the immune system in the development of the disease induced by the *Premolis semirufa* caterpillar, this study aimed to evaluate the phenotype of the immunological response induced by repeated injections of the caterpillar bristle extract in a murine model.

## Materials and Methods

### Extract of Caterpillar Bristles

Caterpillars from *Premolis semirufa* were collected in non-protected areas of the city of São Francisco do Pará, Pará, Brazil (license for capture, transportation and maintenance of the animals were provided by Chico Mendes Institute for Biodiversity Conservation (ICMBIO) of the Brazilian Ministry of the Environment – permission no. 11971–2) and maintained at the Immunochemistry Laboratory, Butantan Institute, SP, Brazil. The bristle extract was prepared after incubating the caterpillars at 4°C for a few minutes; the bristles were cut off with scissors at the point of insertion in the tegument, avoiding any tegument incision, and then suspended in cold phosphate-buffered saline (PBS) (8.1 mM sodium phosphate, 1.5 mM potassium phosphate, 137 mM sodium chloride and 2.7 mM potassium chloride, pH 7.2). This suspension was macerated with a glass stick, homogenised and centrifuged at 560×*g* for 20 min at 4°C. The supernatant was collected, and its protein content was determined using the BCA Protein Assay Kit (Pierce Biotechnology, Rockford, IL, USA). Supernatant aliquots were stored at −80°C until use. Authorisation to access the venom of the *Premolis semirufa* caterpillar was provided by the Brazilian Institute of Environment and Renewable Natural Resources (IBAMA), an enforcement agency of the Brazilian Ministry of the Environment (permission no. 01/2009).

The concentration of lipopolysaccharides (LPS) in the samples of *Premolis semirufa* bristle extract was evaluated by the Limulus Amebocyte Lysate (LAL) test in the Section of Microbiological Control of the Butantan Institute (Service of Quality Control - Bioindustrial Division), with the PYROGENT™ Plus Gel Clot LAL Assays kit (Lonza, Walkersville, MD, USA), according to the manufacturer's specifications. The concentration of endotoxin, calculated using a standard curve of LPS from *E*. *coli* (2.5 to 0.125 EU/mL), showed values below the limit of detection, *i.e.*, 0.125 EU/mL; thus, all of the effects observed in our experimental model resulted from the components present in the extract.

### Mice and Ethics Statement

BALB/c strain male mice, aged 2 months and weighing 18–22 g, were obtained from Central Animal Breeding from the Butantan Institute, SP, Brazil. All experimental procedures involving animals were in accordance with the ethical principles in animal research adopted by the Brazilian Society of Animal Science and the National Brazilian Legislation no.11.794/08. The protocol was approved by the Animal Care and Use Committee from Butantan Institute (permission no. 413/07).

### Treatment of Mice with *Premolis semirufa* Bristle Extract

BALB/c mice were injected with 50 µL of pyrogen-free saline containing 10 μg (protein) of the extract in the left hind footpad. The control group mice received 50 µL of pyrogen-free saline in the left hind footpad. The animals were injected seven times at intervals of two weeks. Forty-eight hours after the 1^st^, 3^rd^, 5^th^ and 7^th^ extract inoculations, groups of mice were euthanized using carbon dioxide (CO_2_), their blood was collected for flow cytometry analysis, and the left hind footpads were removed and frozen for immunohistochemistry and immunofluorescence analyses. Additional groups of mice were injected with saline or extract as described above, and 48 h after the 1^st^, 4^th^ and 7^th^ extract inoculations, the animals were euthanized, and their left popliteal lymph nodes were collected and processed for flow cytometry analysis. Concomitant groups of mice were euthanized 24 h after the 1^st^, 4^th^ and 7^th^ extract inoculations, and their blood and hind footpads were collected and homogenised for cytokine analysis. As a positive control for the inflammatory reaction in the immunohistochemistry and immunofluorescence analyses, BALB/c mice were subcutaneously injected with 50 µL of pyrogen-free saline containing 200 μg of Carrageenan (Sigma Chemical CO, St Louis, MO, USA) into the hind footpad. Carrageenan is a polysaccharide widely used to induce an acute inflammatory reaction in animals because it causes the release of several inflammatory mediators such as histamine and prostaglandins [15].

### Antibodies and Flow Cytometry

Flow cytometric analysis was performed on blood samples (25 μL/well) incubated with previously titrated antibodies for 30 min at room temperature (RT). Erythrocytes were lysed using BD FACS™ *Lysing Solution* (BD Pharmingen, San Jose, CA, USA), according to the manufacturer's instructions. Samples were resuspended in 400 μL of FACS buffer (1% BSA and 0.01% sodium azide in PBS) and analysed by flow cytometry (FACSCalibur - Becton Dickinson, San Jose, CA, USA).

The left hind paw popliteal lymph nodes were macerated to obtain cell populations in suspension. The total number of viable cells obtained from each group was determined by counting in a Neubauer chamber in the presence of Trypan blue, and the concentrations were adjusted to 1×10^5^ cells/25 μL of sample in FACS buffer. The cells were incubated for 30 min at 4°C with 5% mouse serum to prevent non-specific binding via the Fc receptor. After removal of the blocking solution, the cells were incubated with previously titrated antibodies for 1 h at 4°C. Following cell staining, the samples were fixed with 1% buffered paraformaldehyde (400 μL) prior to analysis by flow cytometry (FACSCanto II - Becton Dickinson, San Jose, CA, USA).

The following antibodies were used for cell surface staining and were purchased from BD Pharmingen (San Jose, CA, USA): anti-mouse CD3 PE-Cy5 or PE (clone 17A2; IgG2b rat; diluted 1∶400), anti-mouse CD4 FITC (clone GK1.5; IgG2b rat; diluted 1∶400) or anti-mouse CD4 PerCP (clone RM4–5; IgG2a rat; diluted 1∶400), anti-mouse CD8 PE (clone 53–6.7; IgG2a rat; diluted 1∶200), anti-mouse CD19 FITC (clone 1D3; IgG2a rat; diluted 1∶50), anti-mouse CD25 APC (clone PC61; IgG1 rat; diluted 1∶400), anti-mouse CD28 PE (clone 37.51; IgG2 hamster; diluted 1∶200), anti-mouse CD40 PE (clone 3/23; IgG2a rat; diluted 1∶133), anti-mouse CD44 PE (clone IM7; IgG2b rat; diluted 1∶200), anti-mouse CD80 PE (clone 16–10A1; IgG2 hamster; diluted 1∶100), anti-mouse CD86 PE (clone GL1; IgG2a rat; diluted 1∶133), anti-mouse CD154 PE (clone MR1; IgG3 hamster; diluted 1∶200), anti-mouse MHC II PE (clone M5/114.15.2; IgG2b rat; diluted 1∶133) and anti-mouse IL-17R APC (clone PAJ-17R; IgG2a rat; diluted 1∶133). Isotype-matched, non-specific controls were assayed in parallel (BD Pharmingen, San Jose, CA, USA).

Intracellular staining was performed to detect IL-17 and Foxp3. After surface staining, the fixed cells were permeabilized by incubation in 0.2% Triton X-100 (Sigma-Aldrich, St. Louis, MO, USA) in PBS for 6 min at RT, followed by staining with anti-IL-17A-PE (clone 17B7; IgG2a rat; diluted 1∶100) or anti-mouse Foxp3 Alexa Fluor 488 (clone MF23; IgG2b rat; diluted 1∶100).

The expression of specific cell surface markers was analysed on histogram plots, after gating the lymphocyte, granulocyte or monocytes populations on FSC x SSC dot plots. The results of the flow cytometry were expressed as the absolute number and median fluorescence intensity (MFI) of cells positive for the molecules under study.

### Immunohistochemistry and Immunofluorescence Staining

The left hind footpads were removed and frozen in base moulds filled with frozen tissue matrix O.C.T. (Tissue-Tek® O.C.T. Compound, Sakura) and stored at −80°C until sectioning. The sections were cut to a 5 µm thickness and fixed by immersion in cold acetone (−20°C). After the samples were incubated with blocking buffer Protein Block Serum-Free (Dako North America, Carpinteria, CA, USA) to block non-specific binding, the tissue sections on the slides were incubated with purified primary antibody diluted in 2% foetal bovine serum (FBS) in PBS for 2 h at 37°C in a humidified chamber. Following blocking, endogenous peroxidase activity (Dako North America, Carpinteria, CA, USA), endogenous avidin and biotin were blocked with the Avidin*-*Biotin Blocking kit (Biocare Medical, Concord, CA, USA), and then the slides were incubated with the biotinylated secondary antibody, diluted 1∶100, in PBS for 45 min at RT. The slides were rinsed 2× in PBS, 5 min each time, and incubated with Streptavidin-Horseradish Peroxidase (BD Pharmingen, San Jose, CA, USA) for 30 min at RT. After the slides were rinsed 2× in PBS, for 5 min per rinse, 3, 3′-diaminobenzidine, DAB substrate solution (BD Pharmingen, San Jose, CA, USA) was added, and the reaction was followed for 5 to 15 min or until the desired colour intensity was reached. The slides were washed in PBS for 5 min, and then they were counterstained with Mayer's haematoxylin (Sigma-Aldrich, Missouri, USA) and mounted in Kaiser's glycerol gelatine (Merck, Darmstadt, HE, DE).

In the immunohistochemical analysis, the following primary antibodies were used to characterise the local immune response: CD4 (IgG2a rat; diluted 1∶75), CD8 (IgG2a rat; diluted 1∶75), CD11b (IgG2b rat; diluted 1∶25), CD11c (IgG1 hamster; diluted 1∶100), Ly6G (IgG2b rat; diluted 1∶50) and CCR3 (IgG rabbit; diluted 1∶100). Isotype-matched, non-specific controls were assayed in parallel (BD Pharmingen, San Jose, CA, USA).

Alternatively, the tissue sections treated with the purified primary antibody were incubated with the fluorochrome-conjugated secondary antibody diluted in PBS/BSA 1% for 1 h at RT in the dark. After additional washing steps, the sections were counterstained with ProLong Gold antifade reagent with 4,6-diamidino-2-phenylindole (DAPI) (Invitrogen, Eugene, OR, USA). The following fluorochrome-conjugated secondary antibodies were used: TRITC-conjugated goat anti-Rat IgG (dilution 1∶50), FITC-conjugated goat anti-Armenian Hamster IgG (dilution 1∶500) and FITC-conjugated goat anti-Rabbit IgG (dilution 1∶500) purchased from Abcam (Cambridge, UK). Isotype-matched, non-specific controls were assayed in parallel (BD Pharmingen, San Jose, CA, USA).

### Measurement of Cytokines Concentrations in Serum and Mouse Paws

Twenty-four hours after the 1^st^, 4^th^ and 7^th^ extract or saline inoculation, groups of mice were euthanized, and blood samples were collected for cytokine analysis. Bleeding was performed by retro-orbital plexus with a Pasteur pipette. The blood was allowed to clot at RT for 15 min and then kept at 4°C for 6 h. After centrifugation at 560×*g* for 15 min at 4°C, the serum samples were collected and immediately frozen at −80°C until use.

In addition, the hind footpads of mice were collected and homogenised for cytokine analysis. The hind footpads, immersed in ice-cold phosphate buffered saline (PBS) supplemented with the Complete Protease Inhibitor Cocktail Set (Roche Diagnostics, Mannheim, DE), 1 mL/paw, were cut off, frozen in dry ice, and subsequently homogenised using a PT 10–35 Polytron homogeniser (Kinematica, Luzern, SWZ), as described by Okumura (2008) [16], with slight modifications. After homogenisation, the samples were centrifuged at 4°C for 2,195×*g* for 15 min; then, the supernatants were re-centrifuged at 4°C at maximum speed, 15,142×*g*, for 15 min. The supernatants were carefully removed to avoid collecting the top layer of lipids/adipose debris. The supernatants were immediately frozen at −80°C until use.

The sera and the supernatants were then assayed for murine IL-1, IL-2, IL-4, IL-6, IL-10, IL-12, TNF-α, IFN-γ (OptEIA ELISA; BD Pharmingen, San Jose, CA, USA), IL-17 and IL-23 (eBioscience, San Diego, CA, USA), according to the manufacturer's instructions.

### Statistical Analyses

Student's t-test was used to compare mean values obtained with the saline group and the mean values of the *P*. *semirufa* group, and two-way ANOVA with Bonferroni post-tests were used to evaluate significant differences between the inoculations. Statistical analysis was performed using GraphPad Prism software. Differences were considered statistically significant when *p* values were *p*<0.05, *p*<0.01 and *p*<0.001.

## Results

### T and B Lymphocytes are activated by *Premolis semirufa* Bristle Extract

**Figure 1:**
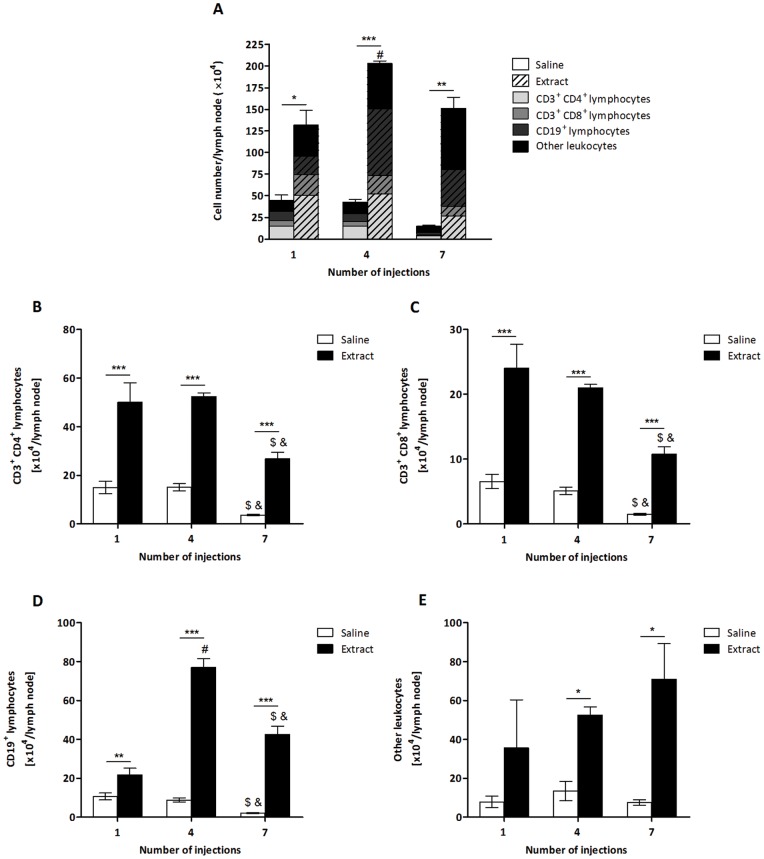
Determination of the total number of leukocytes from *P. semirufa* bristle extract-treated group. BALB/c mice were repeatedly injected with 50 µL of pyrogen-free saline with or without 10 μg (protein) of the extract in the footpad and, after the 1^st^, 4^th^ and 7^th^ inoculations, the popliteal lymph nodes were collected and processed for flow cytometry analysis. (A) Total number of cells, (B) Total number of TCD4 lymphocytes, (C) Total number of TCD8 lymphocytes, (D) Total number of B lymphocytes and (E) Total number of other leukocytes. All graphs show mean values ± SD. *p<0.05, ** *p*<0.01 and ***p<0.001: significant differences between the mean values obtained with the saline group and the mean values of the *P*. *semirufa* group. The symbols indicate significant differences between the inoculations: 1^st^×4^th^ (#), 1^st^×7^th^ (&) and 4^th^×7^th^ ($).

To characterise the immune response to *P. semirufa*, BALB/c mice were repeatedly inoculated with *P. semirufa* bristle extract or sterile saline (control) and the cells from the popliteal lymph nodes collected after the 1^st^, 4^th^ and 7^th^ injections. At the three time points analysed, the total number of cells was significantly higher in mice injected with the extract than in the control animals (Figure 1A), demonstrating the proliferation/migration of immune cells, which reached the highest number after the 4^th^ injection (Figure 1A).

To determine which cell populations were involved in the immune response to *P. semirufa* in this model, lymph node cells were stained with fluorochrome-conjugated mAbs for the detection of T and B lymphocytes by flow cytometry. The absolute number of TCD4, TCD8 and B lymphocytes was significantly higher in the extract-treated group than in the control group in all of the periods analysed (Figure 1B to D). The number of other leukocytes was also increased after the 4^th^ and 7^th^ injections (Figure 1E). Interestingly, the increase in the TCD4 population was more pronounced than that of the B population after the 1^st^ injection, while the number of B cells increased more after the 4^th^ and 7^th^ injections (Figure 1A); thus, the T cells were activated earlier by the *P. semirufa* bristle extract, while the proliferation and/or recruitment of B cells occurred later.

In addition, we observed that there was a decrease in the number of TCD4 and TCD8 lymphocytes in both groups after the 7^th^ injection (Figure 1B and C). This also occurred with the B cells in the control group, although the number of B cells after the 7^th^ injection was lower than after the 4^th^ injection but higher than after the 1^st^ injection in the extract-treated group (Figure 1D), corroborating the late proliferation and/or recruitment of B cells by the *P. semirufa* bristle extract.

**Figure 2:**
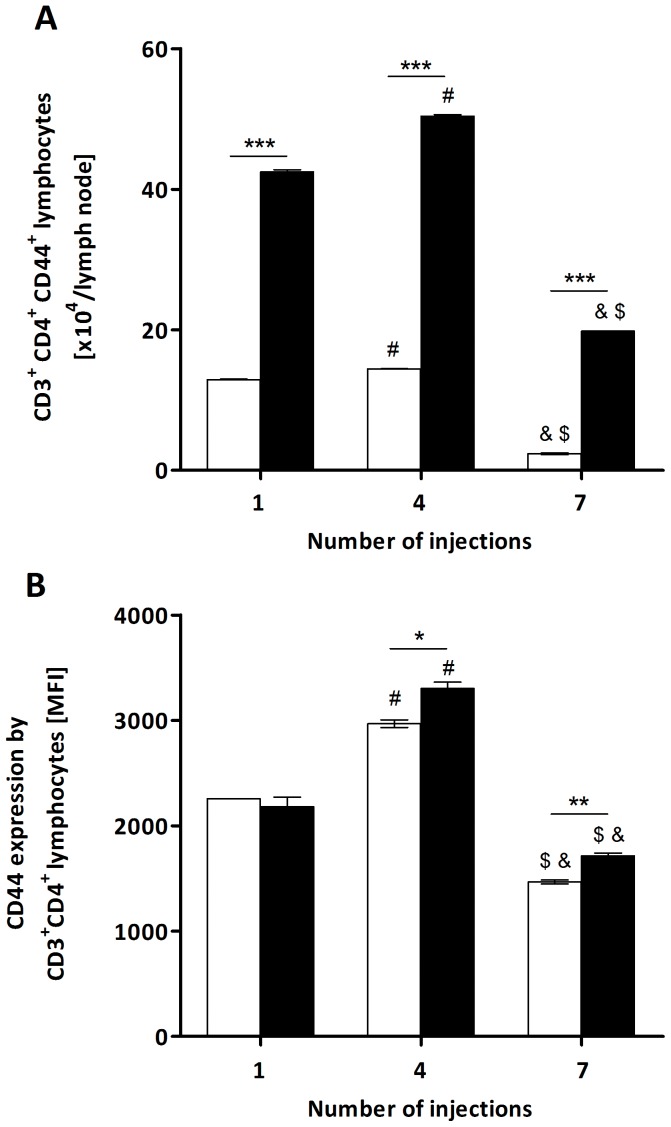
Total number of CD3^+^CD4^+^CD44^+^ T cells and CD44 expression from *P. semirufa* group. BALB/c mice were repeatedly injected with 50 µL of pyrogen-free saline (□) or 10 μg (protein) of the extract (▪) into the footpad and, after the 1^st^, 4^th^ and 7^th^ inoculations, the popliteal lymph nodes were collected and processed for flow cytometry analysis. (A) Total number of CD3^+^CD4^+^CD44^+^ T lymphocytes and (B) Median Fluorescence Intensity (MFI) of the expression of this molecule. All graphs show mean values ± SD. *p<0.05, ** *p*<0.01 and ***p<0.001: significant differences between the mean values obtained with the saline group and the mean values of the *P*. *semirufa* group. The symbols indicate significant differences between the inoculations: 1^st^×4^th^ (#), 1^st^×7^th^ (&) and 4^th^×7^th^ ($).

To further demonstrate the activation of T and B lymphocytes by the extract, these cells' expression of activation molecules was analysed by flow cytometry. Similar to the observation for total TCD4 lymphocytes (Figure 1B), the number of these cells expressing CD44 increased in treated mice compared to the control group at the three points tested (Figure 2A). We also verified a significant increase in the expression level (MFI) of this molecule after the 4^th^ and 7^th^ injections in the extract-treated group compared to the control (Figure 2B). These results indicate the activation of TCD4 cells by the *P. semirufa* bristle extract.

The levels of expression of CD28 and CD154 by TCD4 lymphocytes were also analysed, but no differences were observed between the treated and control groups. Additionally, no TCD4 lymphocytes positive for Foxp3 (Treg) were found in any period analysed (data not shown).

**Figure 3:**
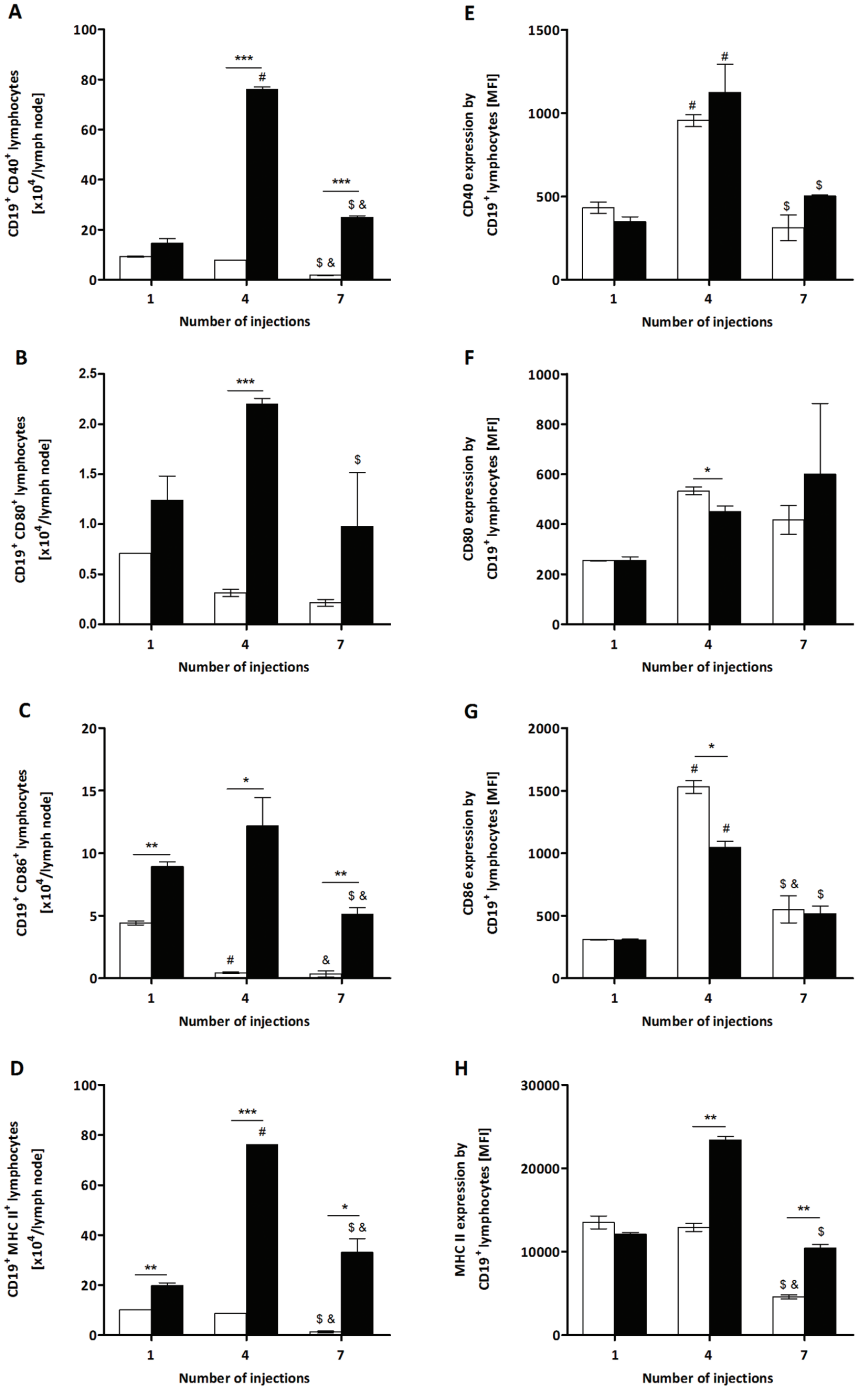
Total number of CD40^+^, CD80^+^, CD86^+^ and MHC II^+^ B cells and their expression from *P. semirufa* group. BALB/c mice were repeatedly injected with 50 µL of pyrogen-free saline (□) or 10 μg (protein) of the extract (▪) in the footpad and, after the 1^st^, 4^th^ and 7^th^ inoculations, the popliteal lymph nodes were collected and processed for flow cytometry analysis. Total number of (A) CD19^+^CD40^+^ B lymphocytes, (B) CD19^+^CD80^+^ B lymphocytes, (C) CD19^+^CD86^+^ B lymphocytes and (D) CD19^+^MHC II^+^ B lymphocytes. Expression of these molecules (MFI) is shown in panels E to H. All graphs show mean values ± SD. *p<0.05, ** *p*<0.01 and ***p<0.0001: significant differences between the mean values obtained with the saline group and the mean values of the *P*. *semirufa* group. The symbols indicate significant differences between the inoculations: 1^st^×4^th^ (#), 1^st^×7^th^ (&) and 4^th^×7^th^ ($).

The activation state of the B cells was evaluated by the expression of molecules involved in their antigen-presenting function to TCD4 lymphocytes. The absolute number of these cells positive for CD40 was higher in extract-treated mice after the 4^th^ and 7^th^ injections than in the control group (Figure 3A), while the number of B cells expressing CD80 was elevated only after the 4^th^ injection (Figure 3B). The number of B lymphocytes positive for CD86 and MHC II increased in all of the periods analysed (Figure 3C and D). For the four molecules, the highest number of positive cells was attained after the 4^th^ injection with the bristle extract (Figure 3A to D), as observed for total B lymphocytes (Figure 1D). However, a decrease in the expression (MFI) of CD80 and CD86 by B lymphocytes was also observed in mice treated with the extract after the 4^th^ injection (Figure 3F and G), in contrast to the expression of MHC II, which increased after the 4^th^ and 7^th^ inoculations (Figure 3H). There was no difference in the CD40 expression by these cells (Figure 3E). These results indicate that the *P. semirufa* bristle extract was able to up-regulate molecules involved with the antigen-presenting function of B lymphocytes, which occurred later than the activation of TCD4 cells.

**Figure 4:**
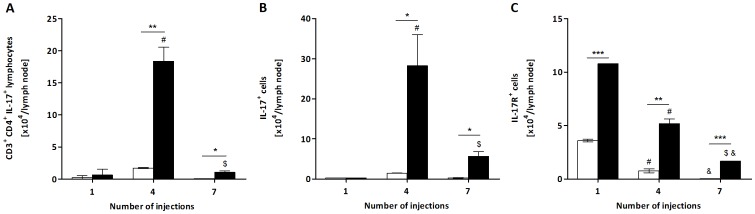
Total number of CD3^+^CD4^+^IL-17^+^ T lymphocytes, IL-17^+^ cells and IL-17R^+^ cells from *P. semirufa* group. BALB/c mice were repeatedly injected with 50 µL of pyrogen-free saline (□) or 10 μg (protein) of the extract (▪) in the footpad and, after the 1^st^, 4^th^ and 7^th^ inoculations, the popliteal lymph nodes were collected and processed for flow cytometry analysis. Total number of (A) CD3^+^CD4^+^IL-17^+^ T lymphocytes, (B) IL-17^+^ cells and (C) IL-17R^+^ cells. All graphs show mean values ± SD. *p<0.05 and ***p<0.0001: significant differences between the mean values obtained with the saline group and the mean values of the *P*. *semirufa* group. The symbols indicate significant differences between the inoculations: 1^st^×4^th^ (#), 1^st^×7^th^ (&) and 4^th^×7^th^ ($).

Another interesting aspect of Pararamose (Pararama-associated phalangeal periarthritis) is its similarity to the clinical signs observed in rheumatoid arthritis. Because IL-17 is an important cytokine involved in the development of rheumatoid arthritis in both humans and the mouse model [17]–[19], its role in the immune response to the *P. semirufa* bristle extract was also examined. IL-17 is generally produced by TCD4 lymphocytes, but other cells, such as γδ T-cells, NK cells (natural killer cells), NK T cells, macrophages, DCs (dendritic cells), neutrophils, mast cells and lymph tissue inducer cells, can also produce this cytokine [20]. In the present work, we determined the number of IL-17-producing TCD4 lymphocytes in the popliteal lymph nodes of control and treated mice, as well as the number of total lymph node cells positive for this cytokine. Interestingly, after the 4^th^ and 7^th^ injections, the number of IL-17^+^ TCD4 lymphocytes was significantly higher in the extract-treated mice than in the control group (Figure 4A). Total lymph node cell analysis led to a similar observation (Figure 4B). Additionally, the number of cells positive for the IL-17 receptor (IL-17R) also increased in the lymph node of the extract-treated group in all of the periods analysed (Figure 4C).

### Effect of the *Premolis semirufa* Bristle Extract on Circulating Leukocytes

The extract decreased the percentage of TCD4 lymphocytes after the 1^st^ injection and increased it after the 3^rd^ injection (Figure S1 A), while the percentage of B lymphocytes was reduced after the 3^rd^ injection and elevated after the 7^th^ injection (Figure S1B). These results suggest that the activation of blood B cells by the extract occurred later than the activation of blood TCD4 lymphocytes. There was no difference in the percentage of TCD8 cells between the treated and control groups (data not shown). In addition to the lymphocyte populations, blood monocytes and neutrophils were also evaluated. The *P. semirufa* bristle extract interfered with the percentage of monocytes at all of the time points analysed, decreasing this percentage after the 1^st^ and 7^th^ injections, but increasing it after the 3^rd^ and 5^th^ injections (Figure S1 C). There was no difference in the percentage of circulating neutrophils between the treated and control groups (data not shown).

The activation status of blood leukocytes was determined by the analysis of the expression of activation molecules by TCD4 lymphocytes, B lymphocytes and monocytes. CD44 expression by blood TCD4 cells showed that they was altered by the *P. semirufa* bristle extract because an increase in the percentage of these cells positive for CD44 after the 5^th^ injection, compared to the control group, was observed (Figure S2 A), as well as higher expression (MFI) of this molecule after the 1^st^ and 3^rd^ injections (Figure S2 B). No differences were observed in the expression of CD28 and CD154 by blood TCD4 lymphocytes between the treated and control groups (data not shown). Blood B lymphocytes were also altered by the *P. semirufa* bristle extract, showing a significant increase in the percentage of these cells positive for CD40 after the 3^rd^ injection (Figure S3 A), as well as for CD80 and MHC II after the 1^st^ injection (Figure S3 B and C). The extract also induced alteration in the expression of molecules in the monocytes population, increasing the percentage of these cells positive for CD86 after the 3^rd^ injection (Figure S3 D), for CD80 after the 5^th^ injection (Figure S3 E) and for MHC II after the 1^st^ injection (Figure S3 F), although the number of CD86^+^ cells decreased after the 7^th^ injection (Figure S3 D). No differences were observed in the expression levels (MFI) of these co-stimulatory molecules by blood B lymphocytes and monocytes (data not shown).

### Local Immune Response Induced by *Premolis semirufa* Bristle Extract

**Figure 5:**
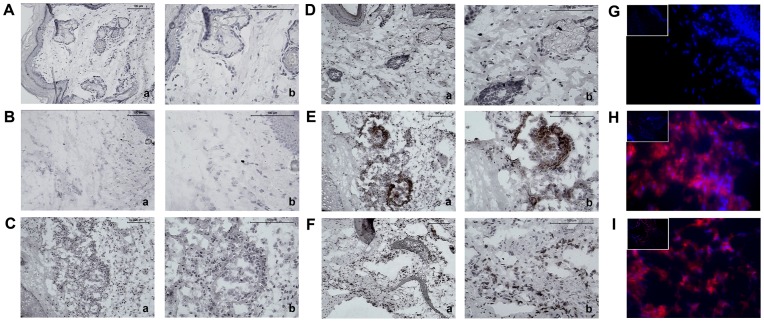
Injection of the *P. semirufa* bristle extract induces neutrophil infiltration at the inoculation site. BALB/c mice were repeatedly injected with 50 µL of pyrogen-free saline with or without 10 μg (protein) of the extract in the footpad and, after the 1^st^, 3^rd^, 5^th^ and 7^th^ inoculations, the footpads were removed and frozen for immunohistochemistry and immunofluorescence analyses. Representative photomicrographs of left hind footpads stained with Ly6G, injected once (**A** and **B**) or seven times (**D** and **E**) with saline and *P. semirufa* bristle extract, respectively. (**C**) Negative control, injected with extract, marked with matched isotype and (**F**) positive control injected with Carrageenan (200 µg/ paw). Positive immunostaining is indicated by a red-brown colour. Images in panel **a** are at 200× magnification, and images in panel **b** are at 400× magnification. Photomicrograph (400× magnification/100× magnification in the left upper quadrant) panels **G** and **H** show immunofluorescence analysis of groups inoculated one and seven times, respectively, with *P*. *semirufa* bristle extract, while panel **I** shows the group inoculated with Carrageenan. DAPI-labelled nuclei appear blue, while the TRITC-labelled target molecules appear red/magenta.

To gain more insight into the phenotype of the local immune response, we have performed immunohistochemistry and immunofluorescence analyses of the cells infiltrating the site of inoculation. Among the antibodies tested, only Ly6G and CD11b staining was detected in the footpads. After the 1^st^ injection, there were no differences between footpads of mice injected with the *P. semirufa* bristle extract and the control group, with absence of staining for Ly6G (Figure 5A, B and G). In contrast, after the 3^rd^, 5^th^ and 7^th^ injections of the extract, we strongly detected cells positive for Ly6G (Figure 5E and H and data not shown), and these were most likely concentrated in the exact location of the injection, below the dermis in the connective tissue. This finding indicates that these cells migrated to the inoculation site, whereas the control group did not present a significant response (Figure 5D and data not shown). The positive control group (inoculated with carrageenan) also showed intense staining for Ly6G, indicative of the presence of neutrophils (Figure 5F and I).

**Figure 6:**
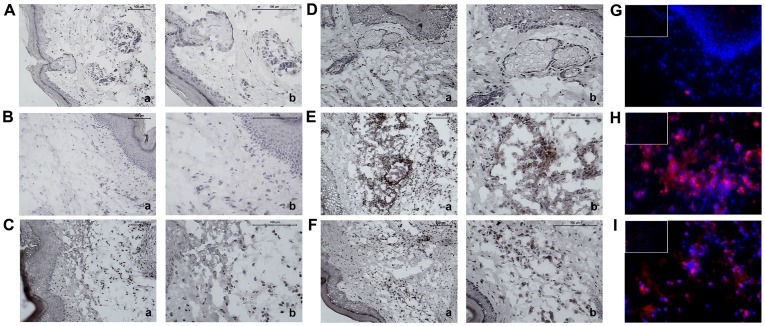
Injection of the *P. semirufa* bristle extract induces the infiltration of macrophages to the inoculation site. BALB/c mice were repeatedly injected with 50 µL of pyrogen-free saline with or without 10 μg (protein) of the extract in the footpad and, after the 1^st^, 3^rd^, 5^th^ and 7^th^ inoculations, the footpads were removed and frozen for immunohistochemistry and immunofluorescence analysis. Representative photomicrographs of left hind footpads stained with CD11b, injected one (**A** and **B**) or seven (**D** and **E**) times with saline and *P. semirufa* bristle extract, respectively. (**C**) Negative control, injected with extract, marked with matched isotype and (**F**) positive control injected with Carrageenan (200 µg/ paw). Positive immunostaining is indicated by the red-brown colour. Images in panel **a** are at 200× magnification, and images in panel **b** are at 400× magnification. Photomicrograph (400× magnification/100× magnification in the left upper quadrant) panels **G** and **H** show the immunofluorescence analysis of groups inoculated one and seven times, respectively, with *P*. *semirufa* bristle extract, while panel **I** shows the group inoculated with Carrageenan. The DAPI-labelled nuclei appear blue, while the TRITC-labelled target molecules appear red/magenta.

There was also a pronounced infiltration of positive CD11b cells in a location close to the marking Ly6G cells (Figure 6E and H), *i.e.*, at the inoculation site, indicating a mixed inflammatory infiltrate composed of neutrophils and macrophages. Again, the group inoculated with saline showed no infiltration of CD11b positive cells (Figure 6A and D).

### Cytokine Profiles in the Serum and Paws from Mice Inoculated with *P*. *semirufa* Bristle Extract

We have previously demonstrated that sera obtained from animals injected with the extract presented higher IgG1 titres than those of other IgG subclasses, suggesting the predominance of a Th2 immune response [14]. Therefore, we sought to determine whether the injection of *Premolis semirufa* bristle extract could elicit that response. Under the conditions and methodologies used, it was not possible to detect the presence of IL-1, IL-2, IL-4, IL-6, IL-10, IL-12, TNF-α, IFN-γ, IL-17 and IL-23 in the sera samples from the inoculated animals, suggesting that *Premolis semirufa* bristle extract causes a local immune reaction rather than a systemic one (data not shown).

**Figure 7:**
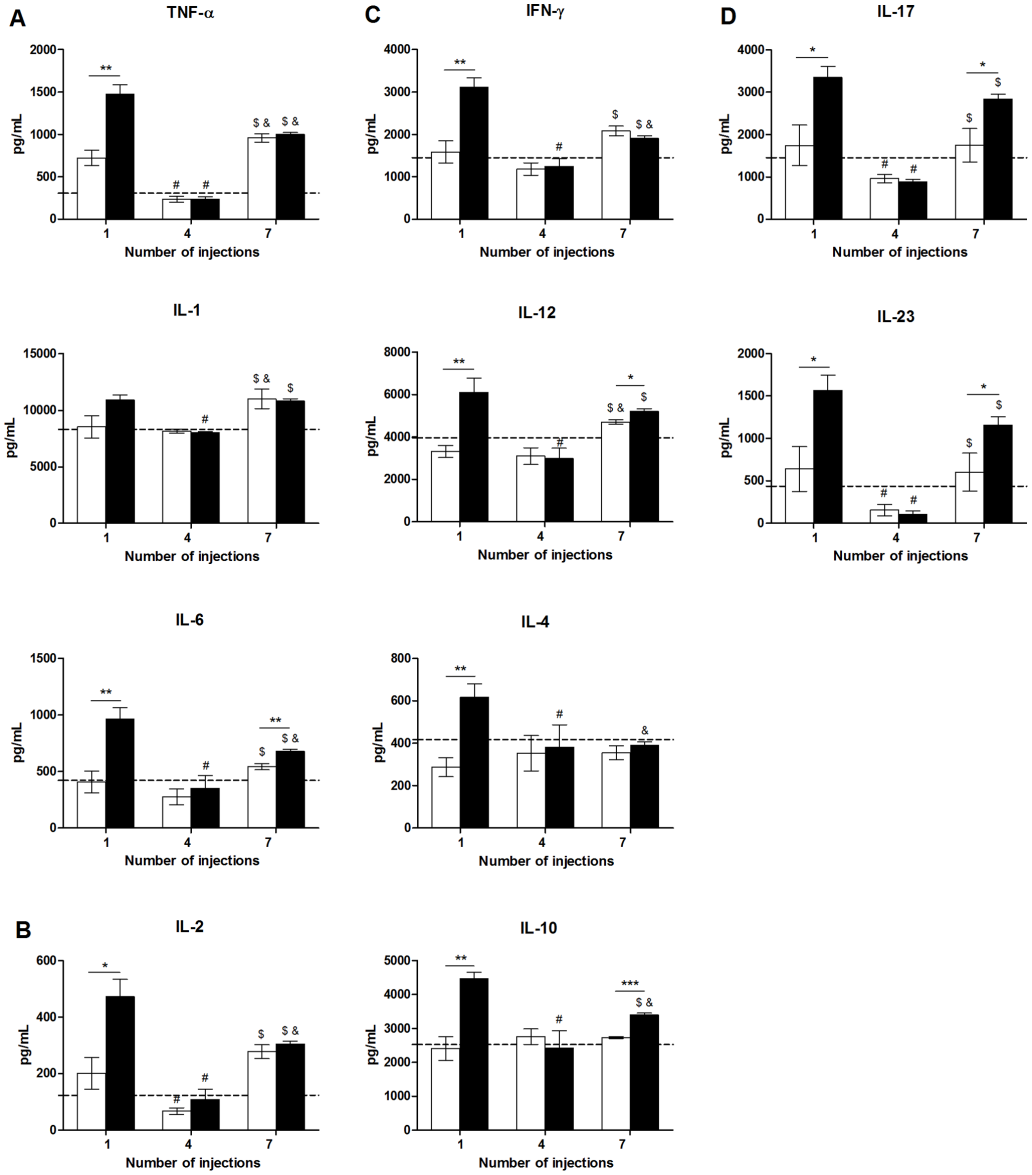
Concentration of paw cytokines from mice injected with *Premolis semirufa* bristle extract. BALB/c mice were repeatedly injected with 50 µL of pyrogen-free saline (□) or 10 μg (protein) of the extract (▪) in the footpad and, after the 1^st^, 4^th^ and 7^th^ inoculations, the footpads were collected and processed for cytokine analysis by ELISA. (A) Levels of innate immunity associated cytokines TNF-α, IL-1 and IL-6, (B) levels of the pro-proliferative cytokine IL-2, (C) levels of T_H_1-associated cytokines IFN-γ and IL-12, T_H_2-associated and anti-inflammatory cytokines IL-4 and IL-10, respectively and (D) levels of T_H_17-associated cytokines IL-17 and IL-23. Un-inoculated paws were used as a control for basal cytokine levels (dotted line). All graphs show mean values ± SD. *p<0.05, ** *p*<0.01 and ***p<0.001: significant differences between the mean values obtained with the saline group and the mean values of the *P*. *semirufa* group. The symbols indicate significant differences between the inoculations: 1^st^×4^th^ (#), 1^st^×7^th^ (&) and 4^th^×7^th^ ($).

Cytokine analysis of the paws showed elevated levels of the innate immunity associated cytokines, such as TNF-α and IL-6, as well as T lymphocyte proliferation associated cytokines, such as IL-2, after the 1^st^ injection of the extract, decreasing after the 4^th^ inoculation (Figure 7A and B). Consistent with marked neutrophil and macrophage infiltration in the mouse paws (Figures 5 and 6E and H), the levels of the pro-inflammatory cytokine IL-6 increased after the 7^th^ injection of the extract, relative to the controls (Figure 7A). Interestingly, the production of IL-1 remained unaltered by the extract-induced inflammatory response.

Similarly, the production of Th1-associated cytokines such as IFN-γ and IL-12, Th2-associated cytokines such as IL-4, and anti-inflammatory cytokines such as IL-10 increased after the 1^st^ inoculation with the extract (relative to the controls) and decreased after the 4^th^ administration of the extract. Curiously, IL-12 and IL-10 levels increased upon the 7^th^ injection of the extract (Figure 7C).

The levels of Th17-associated cytokines, such as IL-17 and IL-23, also markedly increased after the 1^st^ and 7^th^ inoculations with the extract (compared to the control samples) (Figure 7D).

## Discussion

Studies on the pathogenesis of pararama are scarce. Nonetheless, pararamose is a serious problem in occupational medicine and a social problem affecting the Brazilian Amazon region. In the case of chronic envenomation, rubber tappers can no longer return to their activities, source of their livelihood. With the aim of finding experimentally amenable tools for investigation of this medically important disease and reproducing its manifestation, we have developed a mouse model of pararamose. We injected 10 μg (protein) of the extract into the subcutaneous tissue of the hind footpad at intervals of two weeks in an attempt to simulate accidental human contact and to better analyse the possible chronic inflammatory reaction and immune responses at the cellular and humoral levels.

Subcutaneous injection of the *Premolis semirufa* bristle extract caused a significant increase in the total number of cells and of the absolute number of TCD4, TCD8 and B lymphocytes present in the pool of popliteal lymph nodes obtained from groups of inoculated mice after the 1^st^, 4^th^ and 7^th^ inoculations. This high number of cells in the treated group may have been due to migration and/or cellular proliferation induced by the components present in the extract. The fact that the increase in the absolute number of B lymphocytes was more pronounced than the increase in the absolute number of other cell populations after the 4^th^ and 7^th^ injections may indicate an initial proliferation or migration of T lymphocytes being replaced by a later increase in the proliferation or migration of B cells.

When the profile of T and B lymphocyte activation was evaluated, there was an increase in the number of TCD4 lymphocytes expressing the CD44 molecule in the three periods analysed. We also observed a significant increase in the expression levels (MFI) of this molecule after the 4^th^ and 7^th^ injections in the experimental group. CD44, an adhesion molecule expressed on hematopoietic and nonhematopoietic tissues, has been previously recognised as a marker of memory cells and an important receptor for the recruitment of activated T cells, and it acts through interactions with components of the extracellular matrix [21]–[24] and hyaluronic acid (HA) [25], [26]. Thus, the increased number of T lymphocytes expressing CD44 and the increased expression level of this molecule indicates activation of these cells by components of the extract and generation of memory cells, thus contributing to the chronic nature of pararamose.

Among the co-stimulatory molecules expressed on the surface of B cells, CD40 is extremely important and assists in the activation, proliferation, differentiation, survival and generation of memory B cells [27], [28]. Furthermore, CD80 and CD86 play a major role in providing co-stimulation to T cells, leading to their proliferation, the production of cytokines such as IL-2, and the development of effector functions [29], [30]. In our experiments, we observed an increase in the absolute number of positive B cells for CD40, CD80, CD86 and MHC II in extract-treated mice. These findings suggest that activated and proliferating B lymphocytes may be retained in the lymph node, with subsequent differentiation of T cells and immunoglobulin production.

Analysis of TCD4 lymphocytes that express the molecule IL-17 showed that there was an increase in the number of these lymphocytes after the 4^th^ and 7^th^ inoculations with the extract; however, a clear reduction in their number after the 7^th^ inoculation was also observed. IL-17 is a potent pro-inflammatory cytokine produced by T cells (Th17) [31] and other cell types such as γδ T-cells, NK cells (natural killer cells), NK T cells, macrophages, DCs (dendritic cells), neutrophils, mast cells and lymph tissue inducer cells [20]. Recently, it was demonstrated that Th17 lymphocytes are associated with autoimmune diseases, including rheumatoid arthritis, multiple sclerosis and inflammatory bone disease, in humans and mice [32]–[34]. Thus, it is possible to suggest that the extract was able to induce differentiation and proliferation of TCD4 lymphocytes in Th17 lymphocytes, but the role of these cells in pararamose still needs further investigation. The same increase was observed when total lymph node cells were assayed for this molecule, indicating that other cell types also produced this potent pro-inflammatory cytokine during disease progression.

Ultimately, the number of cells positive for the IL-17 receptor (IL-17R) was also elevated in the lymph nodes of the extract-treated mice in all of the periods analysed. The receptor for IL-17 is expressed in many cell types, in which it induces the expression of chemokines, pro-inflammatory cytokines and colony-stimulating factor [35]. These cytokines and chemokines induce the recruitment of neutrophils and other myeloid cells, and this induction is characteristic of many infectious diseases [36]. We hypothesize that, due to its highly inflammatory nature, the increase in the number of cells expressing this receptor may contribute to the genesis of pararamose.

After the 3^rd^, 5^th^ and 7^th^ injections, by immunohistochemical and immunofluorescence methods, it was detected a high concentration of neutrophils in the connective tissue below the dermis, possibly in the region of inoculation. Neutrophils are an essential part of the innate immune system, and they play a key role in the elimination of invading pathogens and the promotion of tissue healing. However, they can also promote persistent inflammatory responses and tissue injury, which can be damaging to the host [37]–[39]. Thus, our results indicate that the components present in the extract promoted a local inflammatory response, and these cells migrated into the inflammatory focus with the purpose of controlling or eliminating the causative factors and promoting tissue healing.

In addition to the influx of neutrophils, *Premolis semirufa* bristle extract induced the recruitment of macrophages to the site of inflammation. At the inflammatory site, macrophages can maintain the inflammatory response by producing cytokines, such as TNF-α, IL-1β and IL-6, which can protect monocytes from apoptosis [40]. Moreover, the absence of lymphocytes in the hind footpads of the mice may indicate a low participation of these cells in the local inflammatory process induced by *Premolis semirufa* bristles extract. The role of activated B and T lymphocytes on the development of pararamose also requires further investigation.

The local reaction 24 h after the 1^st^ inoculation resulted in increased levels in all of the cytokines analysed, including innate immunity associated cytokines (TNF-α, IL-1 and IL-6), T lymphocyte proliferation-associated cytokine (IL-2), Th1-associated cytokines (IFN-γ and IL-12), Th2-associated cytokines (IL-4), anti-inflammatory cytokines (IL-10) and Th17-associated cytokines (IL-17 and IL-23). After the 4^th^ inoculation with the extract, there was a surprising decrease in the levels of all of the locally produced cytokines, phenomenon which requires further investigation. In contrast, the local reaction observed after the 7^th^ inoculation resulted in a significant increase in the levels of cytokines IL-6, IL-12, IL-10, IL-17 and IL-23. These findings suggest that the components present in the extract have pro-inflammatory potential, which is consistent with the neutrophil and macrophage infiltration in the footpads of mice treated with the *P. semirufa* bristles extract.

Among the analysed cytokines, it is interesting to note the presence of IL-17, IL-23 and IL-10. IL-17 and IL-23 are related to the Th17 response, which play important roles in the pathogenesis of several disorders, including inflammatory diseases, autoimmune diseases, and cancers [41]–[43], and this result is in agreement with the presence of IL-17^+^ and IL-17R^+^ cells in the draining lymph node. In addition, IL-23 also has potent effects on cells of the innate immune system, and it induces the production of inflammatory cytokines, such as IL-1, IL-6 and TNF-α by monocytes and macrophages [44]. IL-10 is a well-known anti-inflammatory cytokine that suppresses the synthesis of pro-inflammatory cytokines, such as IL-1, IL-6 and TNF-α [45], and its increase in the footpads of mice treated with *P. semirufa* bristles extract can indicate the attempt to control the strong inflammatory reaction.

In conclusion, data presented here show significant pro-inflammatory changes in the immune phenotype of antigen-presenting cells, lymphocytes, and cytokine production associated with the chronic exposure to *Premolis semirufa* caterpillar bristles extract, which may explain the intense and prolonged inflammatory response that characterises pararamose.

## Supporting Information

Figure S1 **Percentage of leukocytes in the peripheral blood from** ***P*** **.** ***semirufa*** **group.** BALB/c mice were repeatedly injected with 50 µL of pyrogen-free saline (□) or 10 μg (protein) of the extract (▪) in the footpad and, after the 1^st^, 3^rd^, 5^th^ and 7^th^ inoculations, the peripheral blood was collected and processed for flow cytometry analysis. Percentage of (A) CD3^+^CD4^+^ T lymphocytes, (B) CD19^+^ B lymphocytes and (C) CD11b^+^ cells. All graphs show mean values ± SD. *p<0.05, ** *p*<0.01 and ***p<0.0001: significant differences between the mean values obtained with the saline group and the mean values of the *P*. *semirufa* group. The symbols indicate significant differences between the inoculations: 1^st^×3^th^ (#), 1^st^×7^th^ (&), 1^st^×5^th^ ($), 3^th^×5^th^ (α), 3^th^×7^th^ (β) and 5^th^×7^th^ (γ).
(TIF)

Figure S2 **Percentage of CD3^+^CD4^+^CD44^+^ T cells and CD44 expression in the peripheral blood from** ***P*** **.** ***semirufa*** **group.** BALB/c mice were repeatedly injected with 50 µL of pyrogen-free saline (□) or 10 μg (protein) of the extract (▪) in the footpad and, after the 1^st^, 3^rd^, 5^th^ and 7^th^ inoculations, the peripheral blood was collected and processed for flow cytometry analysis. (A) Percentage of CD3^+^CD4^+^CD44^+^ T lymphocytes and (B) Median Fluorescence Intensity (MFI) of the expression of this molecule. All graphs show mean values ± SD. *p<0.05: significant differences between the mean values obtained with the saline group and the mean values of the *P*. *semirufa* group. The symbols indicate significant differences between the inoculations: 1^st^×3^th^ (#), 1^st^×7^th^ (&), 1^st^×5^th^ ($), 3^th^×5^th^ (α), 3^th^×7^th^ (β) and 5^th^×7^th^ (γ).
(TIF)

Figure S3 **Percentage of CD40^+^, CD80^+^, MHC II^+^ B cells and CD80^+^, CD86^+^, MHC II^+^ monocytes from** ***P*** **.** ***semirufa*** **group.** BALB/c mice were injected with 50 µL of pyrogen-free saline (□) or 10 μg (protein) of the extract (▪) in the footpad and, after the 1^st^, 3^rd^, 5^th^ and 7^th^ inoculations, the peripheral blood was collected and processed for flow cytometry analysis. Percentages of (A) CD19^+^CD40^+^ B lymphocytes, (B) CD19^+^CD80^+^ B lymphocytes, (C) CD19^+^MHC II^+^ B lymphocytes, (D) CD11b^+^CD86^+^ monocytes, (E) CD11b^+^CD80^+^ monocytes and (F) CD11b^+^MHC II^+^ monocytes. All graphs show mean values ± SD. *p<0.05 and ** *p*<0.01: significant differences between the mean values obtained with the saline group and the mean values of the *P*. *semirufa* group. The symbols indicate significant differences between the inoculations: 1^st^×3^th^ (#), 1^st^×7^th^ (&), 1^st^×5^th^ ($), 3^th^×5^th^ (α), 3^th^×7^th^ (β) and 5^th^×7^th^ (γ).
(TIF)
