# Supplementary figures and images for: Characterization of Phenotypes of Immune Cells and Cytokines Associated with Chronic Exposure to Premolis semirufa Caterpillar Bristles Extract
Source: PLoS One. 2013 Sep 4;8(9):e71938. doi: 10.1371/journal.pone.0071938 (PMC3762804; doi:10.1371/journal.pone.0071938)

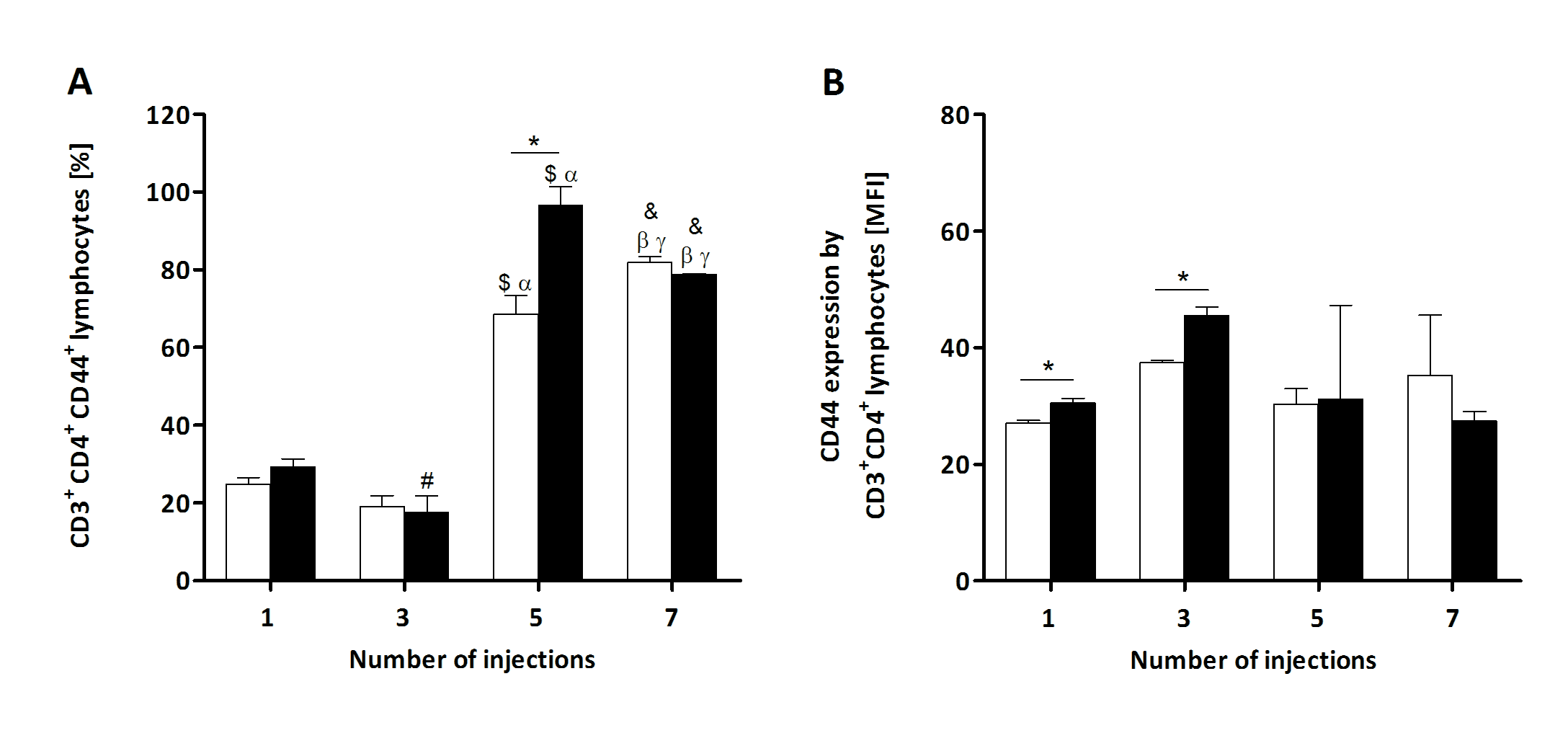

Supplement: Figure S2 — Percentage of CD3+CD4+CD44+ T cells and CD44 expression in the peripheral blood from P . semirufa group. BALB/c mice were repeatedly injected with 50 µL of pyrogen-free saline (□) or 10 μg (protein) of the extract (▪) in the footpad and, after the 1st, 3rd, 5th and 7th inoculations, the peripheral blood was collected and processed for flow cytometry analysis. (A) Percentage of CD3+CD4+CD44+ T lymphocytes and (B) Median Fluorescence Intensity (MFI) of the expression of this molecule. All graphs show mean values ± SD. *p<0.05: significant differences between the mean values obtained with the saline group and the mean values of the P. semirufa group. The symbols indicate significant differences between the inoculations: 1st×3th (#), 1st×7th (&), 1st×5th ($), 3th×5th (α), 3th×7th (β) and 5th×7th (γ). (TIF) [file pone.0071938.s002.tif]

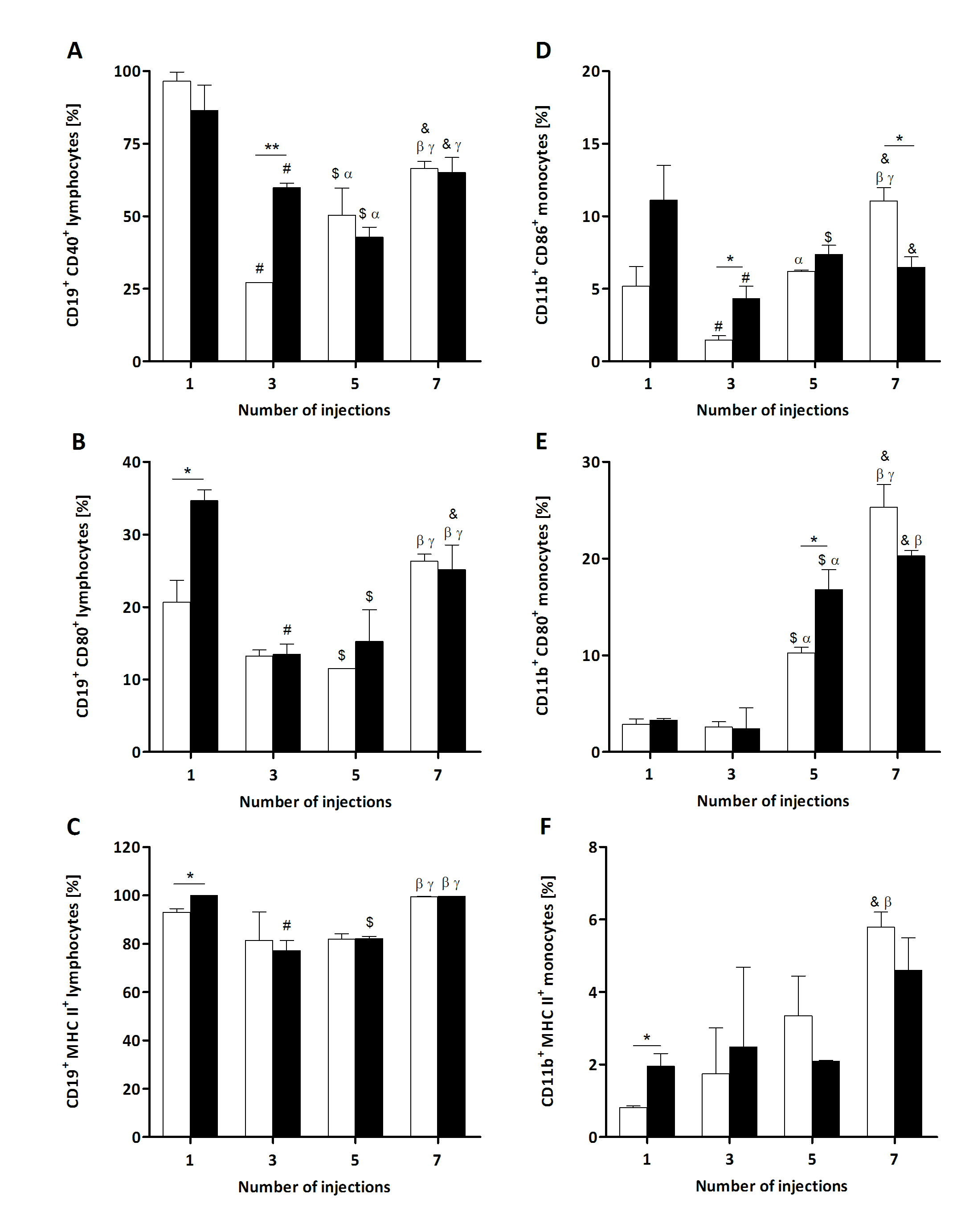

Supplement: Figure S3 — Percentage of CD40+, CD80+, MHC II+ B cells and CD80+, CD86+, MHC II+ monocytes from P . semirufa group. BALB/c mice were injected with 50 µL of pyrogen-free saline (□) or 10 μg (protein) of the extract (▪) in the footpad and, after the 1st, 3rd, 5th and 7th inoculations, the peripheral blood was collected and processed for flow cytometry analysis. Percentages of (A) CD19+CD40+ B lymphocytes, (B) CD19+CD80+ B lymphocytes, (C) CD19+MHC II+ B lymphocytes, (D) CD11b+CD86+ monocytes, (E) CD11b+CD80+ monocytes and (F) CD11b+MHC II+ monocytes. All graphs show mean values ± SD. *p<0.05 and ** p<0.01: significant differences between the mean values obtained with the saline group and the mean values of the P. semirufa group. The symbols indicate significant differences between the inoculations: 1st×3th (#), 1st×7th (&), 1st×5th ($), 3th×5th (α), 3th×7th (β) and 5th×7th (γ). (TIF) [file pone.0071938.s003.tif]
